# Canine visceral leishmaniasis in area with recent *Leishmania* transmission: prevalence, diagnosis, and molecular identification of the infecting species

**DOI:** 10.1590/0037-8682-0141-2020

**Published:** 2020-09-11

**Authors:** Josiane Valadão Lopes, Érika Monteiro Michalsky, Nathália Cristina Lima Pereira, Adão Junior Viana de Paula, Andreza Geisiane Maia Souza, Letícia Cavalari Pinheiro, Ana Cristina Vianna Mariano da Rocha Lima, Daniel Moreira de Avelar, João Carlos França-Silva, Virgínia Aguiar Sorice Lanzetta, Jarbas de Melo, Consuelo Latorre Fortes-Dias, Edelberto Santos Dias

**Affiliations:** 1 Instituto René Rachou, Fundação Oswaldo Cruz Minas, Belo Horizonte, MG, Brasil.; 2 Universidade Federal de Minas Gerais, Departamento de Parasitologia, Belo Horizonte, MG, Brasil.; 3 Prefeitura Municipal de Itaúna, Centro de Controle de Zoonoses, Itaúna, MG, Brasil.; 4 Fundação Ezequiel Dias, Diretoria de Pesquisa e Desenvolvimento, Belo Horizonte, MG, Brasil.

**Keywords:** Leishmaniasis, Visceral leishmaniasis, Leishmania, Canine leishmaniasis

## Abstract

**INTRODUCTION:**

Canine visceral leishmaniasis (CVL) is an endemic disease in Brazil, and integrated control actions have been adopted by the Brazilian Ministry of Health to control its spread. However, the transmission profile is unknown in areas with recent CVL cases, including Itaúna, located in the Brazilian state of Minas Gerais, where the present study was carried out.

**METHODS:**

A total of 2,302 dogs from 12 neighborhoods were serologically tested for canine VL using the current diagnostic protocol adopted by the Brazilian Ministry of Health. Test positivity rate (TPR) and CVL prevalence were determined for each neighborhood. The presence of *Leishmania* was assessed in 60 seropositive dogs which had been recommended for euthanasia. Twenty-two of them (37%) were asymptomatic, and 38 (63%) were symptomatic for CVL. Parasitological (myeloculture and smear/imprint) and molecular (PCR) methods were employed for *Leishmania* detection in bone marrow, spleen, mesenteric lymph nodes, and ear skin. The infecting *Leishmania* species was identified by DNA sequencing.

**RESULTS:**

CVL prevalence (per 1,000 dogs) varied from 0.0-166.67, depending on the neighborhood, with a mean of 68.96 (SD 51.38). *Leishmania* DNA was detected in at least one tissue from all seropositive dogs, with comparable TPR among tissues. *Leishmania* parasites were identified in most (54/60) seropositive dogs, and the infecting parasite was identified as *Leishmania infantum* in all of these.

**CONCLUSIONS:**

Prevalence of CVL is a contributor to the spread of visceral leishmaniasis in Itaúna.

## INTRODUCTION

Visceral leishmaniasis (VL) is a neglected tropical zoonotic disease that is potentially fatal to humans and poses major public health concerns in developing countries. Brazil accounts for approximately 96% of VL cases reported in the Americas^1^. VL is caused by infection with *L. infantum,* and is transmitted through the bite of infected female phlebotomine sand flies; in Brazil, the main vector of *L. infantum* is *Lutzomyia longipalpis* (Diptera: Psychodidae: Phlebotominae). In urban areas, dogs are the most important domestic reservoirs of these parasites, and *Leishmania* amastigotes frequently present in the skin of infected dogs are a source of infection by *L. longipalpis* ^2^ ^-^ ^5^. Canine VL (CVL) has been reported in a number of epidemiological studies in urban areas with active VL transmission. Since CVL frequently precedes the onset of human cases, dogs might be considered sentinel signaling markers of VL^6^ ^-^ ^9^. 

In an attempt to control the increasing geographic expansion and urbanization of VL in Brazil, the Ministry of Health adopted the Surveillance and Control Program of Visceral Leishmaniasis (SCPVL)^10^. The program comprises integrated strategies intended to aid the early diagnosis and treatment of human cases, chemical and environmental control of phlebotomine sand fly vectors, health education, and screening and culling of infected dogs. Based on the average number of human cases (n) reported to the National System of Diseases with Compulsory Notification (SINAN) in the last three years, an epidemiological transmission risk (ETR) score is assigned to different geographical regions, municipalities, or even neighborhoods, as follows: high ETR for n ≥ 4, moderate ETR for 2.4 ≤ n < 4, and sporadic ETR for n < 2.4. SCPVL actions are intensified in areas with high and medium ETRs.

Concerning the screening and culling strategy, every dog presenting positive results by immunochromatography (TR-DPP) testing and enzyme-linked immunosorbent assays (ELISAs) the serial tests currently adopted for CVL diagnosis in Brazil, is considered *Leishmania*-infected and recommended for euthanasia^10^. 

Our study was performed in a Brazilian municipality (Itáuna, Minas Gerais) with recent VL transmission and sporadic ETRs, where systematic control measures have not yet been implemented. In the last few years, however, public health officials from the local Zoonoses Control Center (ZCC) noticed an increase in the number of CVL-positive dogs submitted for serological testing by owners concerned about their dog’s health, termed ‘spontaneous owner’s demand’ (SOD). In a previous study we evaluated parasite-vector relationships through a phlebotomine sand fly survey in Itaúna^11^, due to the lack of local epidemiological data regarding the parasite-vector-reservoir triad involved in the VL transmission cycle. In the present study, we focused on the domestic reservoir, aiming to: a) perform a canine census survey in selected neighborhoods of Itaúna; b) determine the prevalence of CVL in selected neighborhoods using current serological diagnostic tests; c) confirm the presence of *Leishmania* in seropositive dogs using parasitological and molecular-based methods; and d) identify the infecting *Leishmania* species in seropositive dogs.

## METHODS

### Study area

The study was conducted in Itaúna (20**°** 4′ 26″ S, 44° 34′ 24″ W), in the Brazilian state of Minas Gerais, which is an important city in terms of steel and mining activities. Itaúna is located in the center-west region of Minas Gerais, 80 km from the state capital Belo Horizonte. The city occupies an area of 495,769 km^2^ and is divided into 52 neighborhoods. The local population is 92,561 inhabitants in total, according to the latest estimate by the Brazilian Institute of Geography and Statistics^12^.

### Canine samples

Our sample comprised 2,302 dogs, of which 1,690 were from a canine census survey (CCS) performed in 2016 in neighborhoods with previous reports of human and/or canine cases of VL. The canine survey was performed by trained health agents of the local Zoonoses Control Center (ZCC), and included the following neighborhoods: Centro, Chácara do Quitão, Cidade Leonane, Cidade Nova, Garcias, Graças, Morada Nova, Nogueirinha, Novo Horizonte, Olaria, Parque Jardim Santanense, Três Marias, and Vila Nazaré. The remaining dogs (612 animals) were from SOD submissions, in the same year and from the same neighborhoods. 

### CVL diagnosis

All dogs were screened for CVL using the Rapid Dual Path Platform, which detects anti-*Leishmania* antibodies for the *Leishmania donovani* complex via immunochromatography (TR-DPP^®^ LVC, Bio-Manguinhos, Rio de Janeiro, Brazil). The test was performed at the canine owner’s homes after collecting a drop of blood from the ear tip. In cases producing positive results, a new blood sample (3 mL) was collected by puncturing the venal or cephalic vein for examination in a laboratory. The blood serum was separated via centrifugation at 3000 × *g* for 10 min and tested by ELISAs, which quantify anti-*Leishmania* antibodies. In this test, purified proteins from *in vitro* cultures of a *Leishmania major*-like strain (MHOM/BR/71/BH121) were used as antigens (EIE® LVC).

Diagnostic CVL assays were performed by a certified public health institution (Fundação Ezequiel Dias) in accordance with the SCPVL protocol. Dogs with positive immunochromatography and ELISA results were diagnosed as seropositive for CVL and recommended for euthanasia, according to the current policy of the Brazilian Ministry of Health. The owner’s domiciles were georeferenced using a Garmin eTrex hand-held global positioning unit.

### Subsamples of CVL seropositive dogs

CVL-positive dogs (n = 60) were randomly selected for clinical examination by veterinary physicians. A standard form was completed with the dog’s history, including previous vaccinations against rabies and/or VL. Dogs displaying at least one clinical sign attributable to *Leishmania* infection were considered symptomatic for CVL. The clinical signs observed were as follows: lymphadenopathy, corneal opacification, cushion hyperkeratinization, weight loss, ascites, cutaneous alterations (alopecia, furfuraceous eczema, ulcers, snout hyperkeratinization, ear-tip dermatitis), onychogryphosis, keratoconjunctivitis, and hind limb paresis. The affected group was comprised of so-called sick dogs, that is, dogs that presented clinical signs and/or clinicopath abnormalities with confirmed infection^13^. In the absence of any such signs, dogs were diagnosed as asymptomatic for CVL, which corresponded to clinically healthy but infected dogs^13^. Diagnosis was performed as previously described.

### Tissue and bone marrow collection from CVL-seropositive dogs

Dogs were sedated with xylazine (1.1-2.2 mg/kg body weight, given intramuscularly) and anesthetized with thionembutal (adjusted according to animal body weight), before bone marrow aspiration via sterile puncture of the tibial crest. Subsequently, the animals were euthanized using intravenous injection of 0.5 mg/kg of 20% potassium chloride. Biopsy samples were collected from the spleen, mesenteric lymph nodes, and ear skin, and used for preparation of smears or imprints, and for DNA extraction. A total of 240 samples (4 tissue samples per dog, in 60 dogs) were analyzed.

### Investigation of Leishmania parasites

Bone marrow aspirates were seeded into tubes containing NNN (Novy-MacNeal/Nicolle) media enriched with LIT (liver infusion tryptose) and maintained at 25 ± 1°C in an incubator, in duplicate. The myelocultures were examined weekly for the presence of *Leishmania* promastigotes over six weeks (as an indirect parasitological test). The isolates from positive samples were cryopreserved and stored in the strain bank of the Laboratory of Leishmaniases of Instituto René Rachou/Fiocruz Minas. Negative cultures were discarded. Bone marrow aspirates were also used in the preparation of slide smears. Imprints of mesenteric lymph nodes, skin, and spleen were prepared by slide apposition of the respective tissue fragments. After Giemsa staining, slide smear/imprints were examined for the presence of *Leishmania* amastigotes (as a direct parasitological test).

### Investigation of Leishmania DNA

Total DNA was extracted from skin, lymph nodes, and spleen fragments of CVL-seropositive dogs using a Genomic Prep™ Cell and Tissue DNA isolation kit (GE Healthcare, Uppsala, Sweden). GFX^TM^ Genomic Blood DNA Purification (GE Healthcare) was used for DNA extraction from bone marrow aspirates. The procedures were performed according to the manufacturer's instructions. The quality and the mammalian origin of the purified DNA was assessed by PCR amplification of the interphotoreceptor retinoid-binding protein (*IRBP*) gene primed with IRBPfwd (5′-TCCAACACCACCACTGAGATCTGGAC-3′) and IRBPrev (5′-GTGAGGAAGAAATCGGACTGGCC-3′) oligonucleotides^14^. Negative (no DNA) and positive (DNA extracted from dogs not infected by *Leishmania* and living in non-endemic area) control samples were run in parallel. A 227 bp amplified fragment proved the mammal origin of the samples. After DNA assessment, the presence of *Leishmania* DNA was tested by *Leishmania* nested PCR (LnPCR) targeting the small subunit ribosomal RNA (*SSUrRNA*) gene^15^ ^,^ ^16^. In the first amplification step, 10-20 ng of extracted DNA was amplified in the presence of kinetoplastid-specific oligonucleotides using R221 (5′-GGTTCCTTTCCTGATTTACG-3′) and R332 (5′-GGCCGGTAAAGGCCGAATAG-3′) primers. In *Leishmania-*positive samples, a conserved 603 bp fragment was amplified. The PCR products were then diluted 1:40 in sterile water and used as a template in the second amplification step with *Leishmania*-specific primers R223 (5′-TCCCATCGCAACCTCGGTT-3′) and R333 (5′-AAAGCGGGCGCGGTGCTG-3′). Positive samples generated a 358 bp fragment. LnPCR cycling conditions were as previously described^15^ ^,^ ^17^. The amplified products were visualized under UV light after electrophoresis on 2% agarose gels and ethidium bromide staining. Negative (no DNA) and positive (20 ng of DNA extracted from *Leishmania chagasi* - MHOM/BR74/PP75) control samples were used as controls. In all PCR amplifications, we used PureTaq Ready-To-Go PCR Beads (GE Healthcare) in a Veriti 96 well Thermo Cycler (Applied Biosystems, Foster City, USA). 

### Identification of infecting Leishmania species

LnPCR-amplified products were excised and purified from gels using a QIAquick^®^ PCR Purification Kit (Qiagen, Fort Frederick, USA). DNA sequencing was performed using the BigDye^®^ Terminator v3.1 Cycle kit and an ABI 3730 automated DNA sequencing platform (Applied Biosytems) of Instituto René Rachou. Sequencing conditions are described elsewhere^11^. The consensus nucleotide sequence for each sample was aligned and compared to *L. braziliensis* (M80292.1), *L. amazonensis* (M80293.1), and *L. infantum* (M81430.1); the sequences were deposited in the GenBank^®^ database. BioEdit (www.mbio.ncsu.edu/bioedit.bioedit.html), BLAST (www.ncbi.nlm.nih.gov/BLAST), and MacVector^®^ (www.macvector.com, MacVector Inc.) tools were employed for multiple sequence alignments. 

### Statistical and spatial analyses

TPRs for CVL were calculated as follows:

$$TPR=\frac{Number\;of\;seropositive\;dogs\;\times \;100}{Number\;of\;dogs\;tested}$$

Whenever a significant difference was suggested in the TPR data by multiple data comparisons, further analysis was performed in pairs using McNemar’s test with Bonferroni correction. When applicable, result proportions were compared using Fisher’s exact test, with a 95% confidence level (α = 0.05). 

CVL prevalence per neighborhood was determined as follows:

$$Prevalence=\frac{Number\;of\;seropositive\;dogs\;\times \;1000}{Estimated\;canine\;population}$$

The canine population per neighborhood was estimated to be 13.5% of the human population, based on the protocol used in canine anti-rabies vaccination campaigns in the state of Minas Gerais^18^. Human population data are available on the website of the Brazilian Institute of Geography and Statistics^12^. 

Numbers of human VL cases since the first report in 2007 were kindly provided by the Municipal Health Department of Itaúna with respective georeferenced coordinates. *L. longipalpis* and *Leishmania*-infected *L. longipalpis* data were also represented to provide a panorama of VL concerning parasites, vectors, and domestic reservoirs^11^. The R software package was used for map locations^19^. 

### Ethical statements

This study was approved by the Committee on Ethics in Animal Experimentation of the Oswaldo Cruz Foundation (CEUA/Fiocruz) under license no. LW-2/15 (protocol P-68/14-3). The procedures complied with the technical norms established by the Federal Council of Veterinary Medicine (CFMV, Resolution No. 714 of June 20, 2002). 

## RESULTS

### General canine samples

A total of 2,302 dogs were serologically tested for CVL, of which 1,690 were included via CCS and 612 via SOD (Table 1). A total of 358 dogs (21.2%) from the first sample source were positive in the screening test (DPP). Among these animals, CVL diagnosis was confirmed in 203 (56.7%) using ELISA. TPR for CVL was 12.0%, with a mean of 13.2% (SD 7.5%). In the SOD samples, TPR increased to 49.7% (304 of 612 tested using DPP). CVL diagnosis was confirmed in 203 dogs (66.8%) by ELISA. TPRs in the second group were higher (36.5%) than in the first, with a mean of 35.7% (SD 13.8%). Considering both sample sources (CCS plus SOD), TPRs for CVL varied from 10.5-48.0%, depending on the neighborhood, with a mean of 21.3% (SD 11.6%). CVL prevalence varied from 0.0-166.67, according to the neighborhood, with a mean of 68.96 (SD 51.38). SOD dogs were not included in the calculation of CVL prevalence to avoid biasing the results.

**TABLE 1:** Diagnostic test results and calculated prevalence of canine visceral leishmaniasis by Itaúna city neighborhood in the Brazilian state of Minas Gerais, 2016. CCS: canine census survey, SOD: spontaneous owner’s demand. TPR: test positivity rate.

Neighborhood	CCS				SOD				Total (CCS plus SOD)				CVL prevalence^a^
	Dogs	Diagnosis test results		TPR	Dogs	Diagnosis test results		TPR	Dogs	Diagnosis test results		TPR	
	tested (no.)	DPP+	DPP+ ELISA+	(%)	tested (no.)	DPP+	DPP+ELISA +	(%)	tested (no.)	DPP +	DPP+ ELISA+	(%)	
Centro	254	60	21	8.3	61	18	16	26.2	315	78	37	11.7	21.4
Chácara do Quitão	44	10	8	18.2	79	26	19	24.1	123	36	27	22.0	100.0
Cidade Nova	343	64	39	11.4	92	52	26	28.3	435	116	65	14.9	116.8
Cidade Leonane	186	34	19	10.2	27	11	11	40.7	213	45	30	14.1	97.4
Garcias	71	6	5	7	32	28	19	59.4	103	34	24	23.3	13.9
Graças	166	43	23	13.9	25	14	10	40.0	191	57	33	17.3	62.7
Morada Nova	15	0	0	0	83	60	47	56.6	98	60	47	48.0	0.0
Nogueirinha	90	17	15	16.7	4	2	1	25.0	94	19	16	17.0	166.7
Novo Horizonte	143	45	15	10.5	0	0	0	-	143	45	15	10.5	115.4
Olaria	42	9	6	14.3	45	27	11	24.4	87	36	17	19.5	17.1
P. J. Santanense	163	22	16	9.8	116	37	22	19.0	279	59	38	13.6	29.3
Três Marias	154	39	30	19.5	21	8	7	33.3	175	47	37	21.1	104.5
Vila Nazaré	19	9	6	31.6	27	21	14	51.9	46	30	20	43.5	51.3
Total	1690	358	203	12.0	612	304	203	33.2	2302	662	406	17.6	-
Mean ± SD	-	-	-	13.2±7.5	-	-	-	35.7 ±13.8	-	-		21.3±11.6	69.0±51.4

(a) Calculated based on CCS data only.

### Subsample of CVL seropositive dogs

Most seropositive dogs (n = 38, 63.4%) exhibited symptoms of the disease, according to the parameters used for clinical evaluation. The main phenotypic characteristics of the canine subsample are listed in Table 2.

**TABLE 2:** General characteristics of the subsample of CVL-seropositive dogs (n = 60) in an area of recent transmission of visceral leishmaniases. Itaúna, Minas Gerais state, Brazil, 2016.

Variable	Dogs	
	No.	%
Clinical classification		
Asymptomatic	22	36.6
Symptomatic	38	63.4
Gender		
Female	34	56.6
Male	26	43.4
Breed		
Defined 1	16	26.6
Undefined	44	73.4
Hair length		
Short	44	73.4
Long	16	26.6
Size		
Small	21	35.0
Medium	28	46.6
Big	11	18.4

1 Foxhound, Basset Hound, Blue Heeler, Cocker, Pinscher, Poodle, Pit Bull, Fila, Labrador and German Shepherd.

Splenomegaly and lymphadenopathy were observed in 100% of the seropositive dogs. In the symptomatic group, other suggestive clinical signs of CVL included muzzle hyperkeratinization (40%), weight loss (38%), onychogriphosis (37%), keratoconjunctivitis (30%), ear dermatitis (27%), generalized dermatitis (18%), hipped mucosae (5%), decubitus ulcer (3%), localized alopecia (3%), and ascites (2%). (data not shown).

### Detection of Leishmania in subsampled VL-seropositive dogs

**FIGURE 1:**
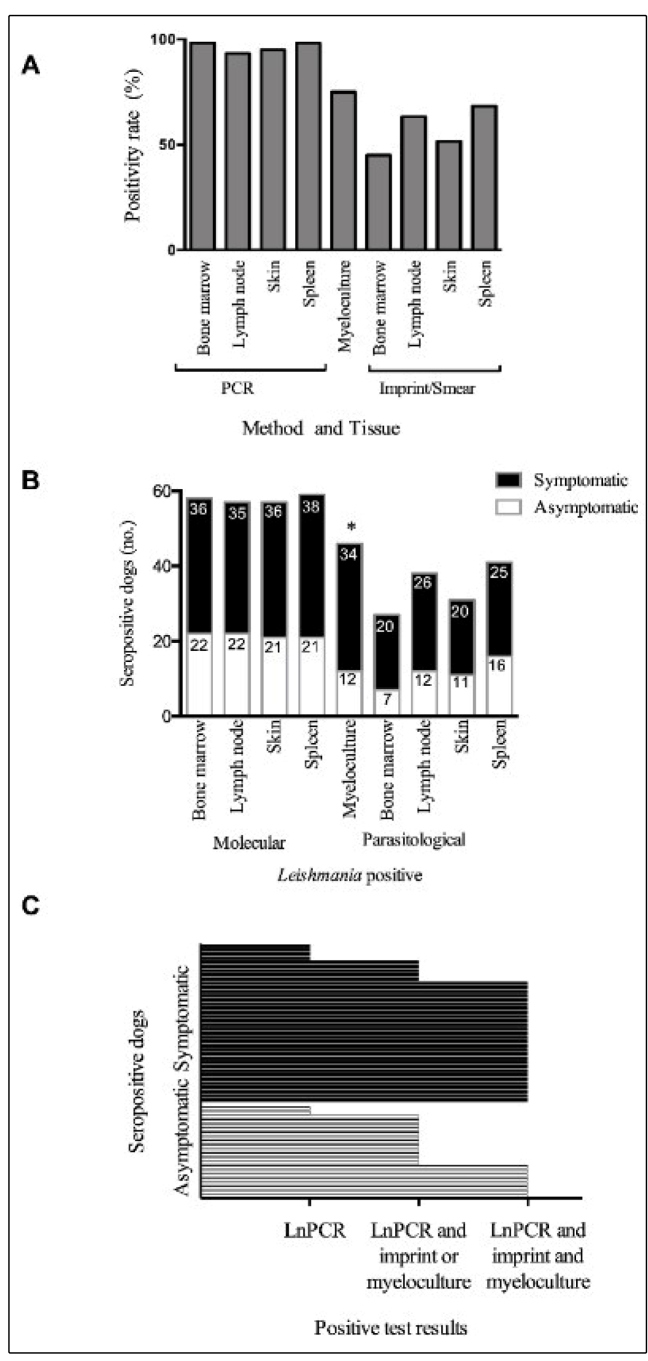
*Leishmania* detection in dogs seropositive for canine visceral leishmaniasis (CVL). (A) Positivity rates of parasitological and molecular tests. (B) Test positivity according to the clinical group. Statistically significant differences between the two groups were observed only for myelocultures (indicated by *) (p < 0.05). (C) Frequency of positive tests according to clinical group. Each horizontal bar represents one dog (n = 60). Itaúna, Minas Gerais state, Brazil, 2016.

The results of molecular and parasitological tests for *Leishmania* in the subsample of dogs seropositive for CVL are presented in Table 3. *Leishmania* DNA was detected in at least one tissue from every dog, including six dogs with negative parasitological results (Table 3). The proportion of positive results for *Leishmania* DNA was comparable for any tissue used for LnPCR (p > 0.05) (Figure 1A). With regard to the presence of *Leishmania* DNA, no statistically significant difference was found between symptomatic and asymptomatic dogs (Figure 1B). 

*Leishmania* promastigotes or amastigotes were identified in every dog except for #23 (asymptomatic) and #24, 42, 47, 56, and #57 (symptomatic) (Table 3). Promastigote forms were detected in 45 of 60 (75%) myelocultures (Figure 1A). *Leishmania* amastigotes were present in 75% of the slide imprints/tissue smears (Figure 1A). Significantly higher TPRs for *Leishmania* amastigotes were obtained for spleen (68.3%) and lymph nodes (63.3%) than for bone marrow (45.0%) or skin (51.7%) samples (adjusted p-values < 0.05) (Figure 1A). TPRs for *Leishmania* amastigotes were significantly higher in the symptomatic group (86.6%) than in the asymptomatic group (54.4%) (p < 0.05) (Figure 1B).

In general, 60% (36/60) of seropositive dogs with CVL had *Leishmania* infection confirmed molecularly (LnPCR for *Leishmania* DNA) and by two parasitological methods, i.e., detection of *Leishmania* promastigotes by myeloculture, and *Leishmania* amastigotes using slide imprints/smears (Figure 1C). *Leishmania* DNA and *Leishmania* parasites (promastigotes or amastigotes) were observed in 18 dogs (30% of the samples). Parasitological tests failed to detect *Leishmania* infection in six dogs (10%), but *Leishmania* DNA was present in their tissues.

**TABLE 3:** Parasitological and molecular test results for *Leishmania* in CVL-seropositive dogs. Itaúna, Minas Gerais state, Brazil, 2016.

Clinical status	Dog no.	Parasitological						Molecular				
			Slide imprint/smear					Bone	Lymph	Spleen	Ski n	Any
		Myeloculture	Bone marrow	Lymph node	Spleen	Skin	Any tissue	marrow	node			tissue
Asymp	4	-	-	+	+	-	+	+	+	+	+	+
	8	-	-	+	+	+	+	+	+	+	+	+
	9	+	+	+	+	+	+	+	+	+	+	+
	10	+	-	-	+	+	+	+	+	+	+	+
	11	-	+	-	+	-	+	+	+	+	+	+
	12	+	+	+	+	+	+	+	+	+	+	+
	15	+	-	-	-	-	-	+	+	+	+	+
	19	+	-	-	-	-	-	+	+	+	+	+
	22	-	-	+	+	+	+	+	+	+	+	+
	23	-	-	-	-	-	-	+	+	+	+	+
	27	-	-	-	+	-	+	+	+	+	+	+
	28	+	-	+	+	+	+	+	+	+	+	+
	32	-	+	+	+	+	+	+	+	+	+	+
	34	+	+	+	+	+	+	+	+	+	+	+
	48	-	-	+	+	-	+	+	+	+	+	+
	49	-	-	+	+	+	+	+	+	+	-	+
	51	+	-	-	-	-	-	+	+	+	+	+
	52	+	+	+	+	+	+	+	+	+	+	+
	53	+	-	-	+	-	+	+	+	+	+	+
	55	+	-	-	-	-	-	+	+	+	+	+
	57	-	-	-	-	-	-	+	+	-	+	+
	60	+	+	+	+	+	+	+	+	+	+	+
Symp	1	+	-	-	-	-	-	+	+	+	+	+
	2	+	-	-	-	-	-	+	+	+	+	+
	3	+	-	+	-	+	+	+	+	+	+	+
	5	+	-	-	-	+	+	+	+	+	+	+
	6	+	+	+	+	+	+	+	+	+	+	+
	7	-	+	+	+	+	+	+	+	+	+	+
	13	+	-	-	-	-	-	+	+	+	+	+
	14	+	-	-	-	-	-	+	+	+	+	+
	16	+	+	+	+	+	+	+	+	+	+	+
	17	+	+	+	-	+	+	+	-	+	+	+
	18	+	+	+	+	-	+	+	+	+	+	+
	20	+	-	-	-	-	-	+	+	+	+	+
	21	+	+	+	+	+	+	+	+	+	-	+
	24	-	-	-	-	-	-	+	+	+	-	+
	25	+	+	+	+	-	+	n.d.	+	+	+	+
	26	+	-	+	+	+	+	+	+	+	+	+
	29	+	+	+	+	+	+	+	+	+	+	+
	30	+	+	+	+	+	+	+	+	+	+	+
	31	+	-	-	+	-	+	+	+	+	+	+
	33	+	+	+	+	-	+	+	+	+	+	+
	35	+	+	+	+	-	+	+	+	+	+	+
	36	+	+	+	-	+	+	+	+	+	+	+
	37	+	-	+	+	+	+	+	+	+	+	+
	38	+	+	+	+	+	+	+	+	+	+	+
	39	+	+	+	+	+	+	+	+	+	+	+
	40	+	+	+	+	+	+	+	+	+	+	+
	41	+	+	+	+	+	+	+	+	+	+	+
	42	-	-	-	-	-	-	+	+	+	+	+
	43	+	+	+	+	+	+	+	-	+	+	+
	44	+	+	+	+	+	+	+	+	+	+	+
	45	+	-	+	+	-	+	+	+	+	+	+
	46	+	-	-	+	-	+	+	+	+	+	+
	47	-	-	-	-	-	-	-	-	+	+	+
	50	+	+	+	+	+	+	+	+	+	+	+
	54	+	+	+	+	+	+	+	+	+	+	+
	56	-	-	-	-	-	-	+	+	+	+	+
	58	+	-	+	+	-	+	+	+	+	+	+
	59	+	-	+	+	-	+	+	-	+	+	+
Positive/total		45/60	27/60	38/60	41/60	31/60	45/60	58/59	56/60	59/60	57/60	60/60

DNA sequencing identified *L. infantum* as the infecting species in 229 of 230 tissue samples containing *Leishmania* DNA. One tissue sample provided inconclusive results in DNA sequencing. 

### CVL prevalence

**FIGURE 2:**
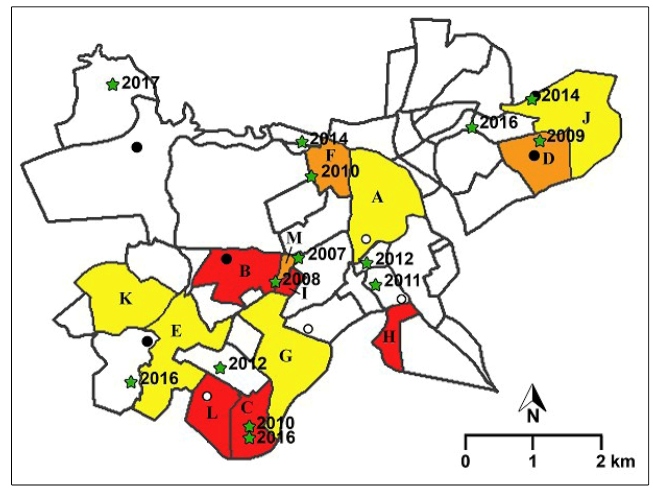
Visceral leishmaniasis scenario in Itaúna (Minas Gerais state, Brazil), an area of sporadic epidemiological transmission risk (ETR) for the disease. Prevalence of canine visceral leishmaniasis (CVL) for each neighborhood is indicated by background color: < 50 (yellow), 50-99 (orange), ≥ 100 (red). A white background indicates non-studied districts. Human visceral leishmaniasis (VL) cases are indicated by green stars and year of report. Sites of *L. longipalpis* capture are represented by circles. Closed circles denote sites of *L. longipalpis* capture which were subsequently found to be infected by *Leishmania* ^11^. Neighborhoods: A. Centro, B. Chácara do Quitão, C. Cidade Nova, D. Cidade Leonane, E. Garcias, F. Graças, G. Morada Nova, H. Nogueirinha, I. Novo Horizonte, J. Olaria, K. Parque Jardim Santanense, L. Três Marias, M. Vila Nazaré.

CVL prevalence per neighborhood was distributed into ranges as follows: < 50, 50-99, ≧ 100 (Figure 2). In an attempt to paint a more complete scenario of urban VL in the city, collection sites of *L. longipalpis* specimens and *Leishmania*-infected *L. longipalpis* (April 2015 to March 2016)^11^, as well as all human VL cases reported since 2007, are also shown in the figure. 

## DISCUSSION

Dogs are the most important reservoirs of *L. infantum* in the domestic infection cycle, and their role in VL transmission in the northeast of Brazil has been recognized since the 1950s. Since then, canine euthanasia has been used as a control measure for VL in an attempt to interrupt the persistence of the parasite in the environment^20^ ^,^ ^21^. Euthanasia is still a major control strategy adopted by SCPVL, although is the least acceptable measure to the population^22^ ^,^ ^23^; the effectiveness of this approach is controversial in the literature. Although there are reports of significant reductions in canine and human VL cases after the removal of seropositive dogs^24^ ^-^ ^26^, other studies have shown that massive euthanasia fail its purpose^22^ ^,^ ^27^ ^-^ ^30^. Mathematical modelling has suggested that culling of symptomatic dogs might be of some help in areas with low transmission rates, even under imperfect conditions (suboptimal screening, diagnosis, and euthanasia rates). Otherwise, the application of strategies integrated together has been suggested as the best choice^22^. Recently, it has been reported that more effective options than dog culling might be on the horizon for VL control in endemic countries^31^.

A major criticism of screening and culling policies, besides the culling of animals itself, is the insufficient sensitivity and specificity of diagnostic tests commonly employed, which lead to false-positive and false-negative results^32^ ^-^ ^35^. A recent study comparing previous (ELISA EIE plus IFI-CVL) and current (TR-DPP and ELISA EIE) diagnostic protocols in Brazil showed an increase in the performance of the latter, and a reduction in false positives^36^. However, a study of 405 asymptomatic dogs from a non-endemic area in Southern Brazil showed no agreement between serological and molecular (real-time PCR) results in cases of low parasite load^37^. In our study, *Leishmania* parasites and/or *Leishmania* DNA were confirmed in 100% of CVL seropositive dogs, regardless of clinical status.

With very few exceptions, *Leishmania* DNA was detected in every tissue from seropositive dogs in our study, with no statistical difference in TPRs between tissues (Table 3, Figure 1A). Using skin DNA as a template for amplification, we obtained a TPR of 95.0% for *Leishmania* DNA. Considering that skin sampling is minimally invasive and collection is simple, this tissue might be suggested for the molecular diagnosis of CVL. However, infected dogs with no lesions in their skins are still able to infect *L. longipalpis* ^38^ ^,^ ^39^. It has been suggested that skin biopsies do not necessarily need to be punched only in lesioned skin, but also in clinically healthy skin for parasite detection^39^.

CVL control is a difficult task. Clinically asymptomatic dogs, and infected dogs with divergent serological results (as shown by parasitological and molecular diagnostic methods), are maintained in people’s homes, where they act as potential sources for *Leishmania* vector infection, and facilitate the disease transmission cycle^4^ ^,^ ^38^ ^-^ ^41^. The operational and ethical implications associated with euthanasia of free-roaming dogs are an additional impediment to CVL control. Interventions promoting responsible pet ownership have been suggested to be an effective strategy^42^. It is important to note that the highest TPR in the screening test (TR-DPP) came from dogs submitted via SOD, compared with CCS canines (Table 1). In addition, all of the positive dogs in the screening test were confirmed to be positive by ELISA. This finding suggested that dog owners are currently attentive to the clinical signs of CVL. However, we cannot discount the possibility of bias in our subsample of seropositive dogs, since most of them were short-haired (Table 2). It has been suggested that this characteristic makes dogs more vulnerable to *Leishmania* infection by making them preferred targets of bites by *Leishmania* vectors^43^ ^,^ ^44^. 

Although our study was restricted to selected neighborhoods, it was possible to confirm favorable conditions for the spread of human VL in Itaúna, such as the concomitant or close presence of a high population of *L. longipalpis, L. infantum*-infected *L. longipalpis,* or *L. infantum-*infected dogs^11^. In a recent ecological study on phlebotomine sand flies^45^, the predominance of *L. longipalpis* among flies captured in Itaúna was confirmed. The species represented 90.4% of the 1,260 specimens captured there. Most importantly, *L. longipalpis* was captured in urban, rural, and forest areas, with a synanthropy index of +95.8. This index measures the degree of association of any given species with a human-modified environment, and varies from -100 (limit of negative association) to +100 (limit of positive association). The value of +95.8 reflects the high adaptation of the *Leishmania* vector in Itaúna to human presence. 

In conclusion, although ETRs have been sporadic until now, the cycle of *Leishmania* transmission is active in Itaúna, and is favored by the presence of parasites, vectors, domestic reservoirs, and human cases of VL. The possibility of applying integrated SCPVL control actions should be evaluated in an attempt to minimize the spread of VL in this and other regions.
